# Supply-demand structural analysis of sports services in Chinese residential care facilities: a cross-sectional study in Zhengzhou

**DOI:** 10.3389/fpubh.2026.1772195

**Published:** 2026-03-19

**Authors:** Shuangrui Liu, Haitao Wang, Yarong Kong

**Affiliations:** College of Physical Education, Kyungpook National University, Daegu, Republic of Korea

**Keywords:** residential care facility, healthy aging, older adults, SERVQUAL, sports service, supply-demand matching

## Abstract

**Background:**

Sports services constitute an important non-medical intervention for promoting healthy aging. China has the world's largest older population with growing demand for institutional care; however, systematic research on supply-demand matching of sports services in residential care facilities remains scarce.

**Methods:**

This cross-sectional study surveyed 278 older adults across 15 residential care facilities in Zhengzhou, China, between March and June 2025. Based on the SERVQUAL gap model, supply-demand status was assessed across five dimensions: facilities, fitness guidance, activity organization, information consultation, and physical monitoring. The dependent variable was constructed using a project counting method: the supply side was measured by service accessibility (SA1–SA5, binary items assessing “whether the institution provides the service”), and the demand side by the number of dimensions with urgent needs (demand mean > 4.0); a difference ≤ 0 was coded as supply deficit. Binary logistic regression was employed with overall satisfaction (*Z*-standardized) as the core independent variable to examine predictors of supply-demand matching. Cronbach's α coefficients ranged from 0.767 to 0.845 across dimensions.

**Results:**

A pronounced structural imbalance in supply and demand was identified. Facilities (gap = 1.03, shortage rate 25.6%) and fitness guidance (gap = 1.02, shortage rate 25.2%) exhibited the strongest demand yet poorest supply, whereas activity organization was relatively well-supplied but ranked lower in demand priority. Significant demographic differences existed across institution types: residents of public institutions had significantly lower self-care ability than those in private institutions (*M* = 1.65 vs. 1.93, *p* = 0.027) and lower monthly income (*M* = 2.17 vs. 2.62, *p* = 0.005); however, overall satisfaction (*p* = 0.695) and supply-demand gap rates (*p* = 0.705) did not differ significantly across institution types. Logistic regression (Nagelkerke *R*^2^ = 0.223, LLR χ^2^ = 49.06, *p* < 0.001) revealed that overall satisfaction (OR = 0.484, *p* < 0.001), education level (OR = 0.612, *p* = 0.002), and monthly income (OR = 0.604, *p* < 0.001) significantly predicted supply-demand matching. After controlling for demographic variables, institution type was non-significant (private OR = 0.801, *p* = 0.500; public-private partnership OR = 0.922, *p* = 0.813), suggesting that inter-institutional differences were primarily attributable to a compositional effect. Among 278 respondents, 97 (34.9%) were classified in the supply-deficit group and 181 (65.1%) in the supply-demand balance group.

**Conclusion:**

Sports services in residential care facilities exhibited a structural contradiction characterized by the coexistence of “high demand-low supply” and “low demand-high supply.” Satisfaction, education level, and monthly income were identified as three key predictors of supply-demand imbalance: older adults with higher satisfaction (i.e., better perceived service quality) had lower odds of supply deficit, while those with higher education and income were associated with less supply-demand mismatch, potentially owing to superior information access and resource utilization capabilities. The non-significant effect of institution type, combined with demographic comparisons, suggested a compositional effect—public institutions bore greater responsibility for functionally impaired and economically vulnerable older adults, yet this structural difference in resident composition was not associated with significant differences in sports service supply-demand matching. However, given the cross-sectional design, these associations should not be interpreted as causal relationships.

## 1 Introduction

Global population aging is advancing at an unprecedented pace. According to the United Nations, the global population aged 60 and above reached 1.2 billion in 2024 and is projected to increase to 2.1 billion by 2050 (1). China's aging process has been particularly rapid: by the end of 2024, the population aged 60 and above had reached 310 million, accounting for 22.0% of the total population (2). The country had 406,000 residential care facilities of various types, housing 2.307 million older residents (3). The older population is projected to exceed 400 million by 2035, with the aging rate surpassing 30% (4). The World Health Organization's “Active Ageing” framework positions physical activity at its core, emphasizing the critical role of exercise in maintaining functional independence among older adults (5). Substantial empirical evidence demonstrates that physical exercise, as an effective non-medical intervention, can significantly improve physical function, cognitive ability, and mental health in older adults (6). However, the development of sports services has lagged considerably behind the rapid expansion of institutional care for older adults, with prevalent deficiencies in facilities, guidance, and service diversity (7). A notable gap persists between national policy mandates and the growing fitness needs of older adults (8, 9). The supply-demand dynamics of sports services in institutional care—a rapidly expanding model of older adults care—have yet to receive adequate scrutiny.

Residential care facilities represent a fundamentally distinct service context from community settings, and the unique characteristics of their residents determine that the nature of supply-demand contradictions differs qualitatively from those in community environments. On the demand side, institutional residents typically present with advanced age, multi-morbidity, and functional dependence, resulting in more complex and diversified sports service needs (10). While community-dwelling older adults largely retain the capacity for autonomous physical activity, institutional residents—given their higher degrees of functional dependence—have more pressing needs for personalized and safety-enhanced exercise guidance (11). On the supply side, the enclosed institutional environment constrains residents' freedom to independently select modes of physical activity, and a care philosophy that prioritizes custodial care over rehabilitation further compresses the space for physical activity (7). Research indicates that older adults in residential care facilities spend approximately 79% of their waking hours in sedentary behavior, with only about 1% devoted to moderate-to-vigorous physical activity (12, 13). Consequently, whereas the core challenge for community-based sports services lies in accessibility, the primary challenge confronting institutional settings is how to deliver effective services to health-vulnerable populations while ensuring safety; the health vulnerability of residents demands exercise programs with enhanced risk management capabilities (14–16). Supply-demand matching conclusions derived from community settings cannot be directly extrapolated to institutional contexts.

The effective provision of public services hinges on structural matching between supply and demand (17). The SERVQUAL model diagnoses service quality issues by comparing the gap between service expectations and perceived performance, and its multi-dimensional gap diagnostic function provides a systematic tool for identifying weaknesses in service provision (18). International experience indicates that effective institutional sports services depend on institutionalized integration: Japan has incorporated exercise rehabilitation into its long-term care insurance system and established a “Functional Training Instructor” system, while the United States has implemented a “Function-Focused Care” model that integrates physical activity into routine care processes (19, 20). These experiences suggest that identifying weaknesses in supply-demand matching is a prerequisite for service optimization. However, although existing Chinese research has revealed widespread supply-demand mismatches in community-based sports services for older adults, given the systematic differences between institutional residents and community-dwelling older adults in health status, autonomy, and environmental constraints, the applicability of these findings to institutional settings remains to be tested (8, 9). Research specifically targeting sports service supply-demand matching in residential care facilities is conspicuously absent, constituting the entry point for the present study.

Based on the aforementioned research gaps, this study systematically analyzes the supply-demand structure and influencing factors of sports services in residential care facilities, aiming to address four questions:

(1) What is the demand profile for sports services among older adults in residential care facilities?
(2) What structural deviations exist in supply-demand matching across dimensions?
(3) What are the key factors influencing supply-demand matching?
(4) How can resource allocation be optimized based on empirical findings?

The theoretical contribution of this study lies in extending the supply-demand matching framework to the domain of institutional sports services, complementing existing research that has predominantly focused on community settings. At the practical level, the findings provide an empirical basis for optimizing sports service resource allocation in older adults care institutions.

## 2 Methods

### 2.1 Study design and theoretical framework

This study employed a cross-sectional design based on the SERVQUAL service quality gap model. The SERVQUAL model, proposed by Parasuraman, Zeithaml, and Berry in 1988, is currently the most widely applied service quality assessment tool. Its core logic involves indirectly measuring service provision levels through customers' perceptual evaluations of actual services.

Satisfaction was adopted as the measure of perceived supply in this study, a choice justified on the following grounds. From a methodological perspective, the SERVQUAL model itself is built upon the “expectation-perception” paradigm (18). From a measurement validity perspective, public service research has widely adopted subjective perception as a core measure of service quality (21).

The core concepts of the SERVQUAL model were operationalized as follows: demand intensity represents older adults' expected level of sports services, satisfaction represents older adults' perceived level of actual service supply, and the difference between them represents the supply-demand gap. This operationalization approach has been widely adopted in public service evaluation research (22). Based on service content attributes, this study measured sports services in residential care facilities across five dimensions: facilities, fitness guidance, activity organization, information consultation, and physical monitoring. This dimensional classification referenced the common categorization in domestic and international sports service research for older adults (9, 23), with adaptive adjustments made for the special characteristics of residential care facility services.

### 2.2 Study area and sampling design

Zhengzhou, as the capital of Henan Province and an important city in central China, has a permanent population exceeding 12.6 million, with 18.1% aged 60 and above. The aging level is slightly below the national average but growing rapidly. As of 2024, the city has over 200 residential care facilities, including approximately 100 comprehensive residential care facilities (providing daily care, medical nursing, and rehabilitation services), covering public, private, and public-private partnership (PPP) types, representing relatively mature institutional care for older adults development and good regional representativeness.

A multi-stage stratified random sampling design was employed to ensure sample representativeness. The first stage stratified by administrative district, selecting five core urban districts (Zhongyuan, Erqi, Guancheng, Jinshui, and Huiji) as the sampling frame; these districts are the primary distribution areas for comprehensive residential care facilities, encompassing approximately 85% of institutional care beds citywide. The second stage stratified by institution type (public, private, PPP); the third stage stratified by institution size (small: <100 beds; medium: 100–200 beds; large: >200 beds), following the Ministry of Civil Affairs' statistical categorization method (24). Computer-generated random numbers were used for random sampling within each stratum: each eligible institution was first assigned a unique identifier, followed by random number sequence generation using SPSS software. Through proportional allocation across strata, 15 comprehensive residential care facilities were randomly selected from 92 eligible institutions in Zhengzhou. The final sample comprised six public institutions, five private institutions, and four PPP institutions.

Participant inclusion criteria were: (1) aged ≥60 years; (2) residence duration ≥3 months, with basic adaptation to institutional life; (3) normal cognitive function, with Mini-Mental State Examination (MMSE) score ≥24; (4) ability to understand and respond to questionnaire items; and (5) voluntary participation with signed informed consent. Exclusion criteria included: (1) severe visual or hearing impairment precluding questionnaire completion; (2) severe mental illness; and (3) physical condition unsuitable for participation during the survey period.

### 2.3 Data collection

Data were collected between March and June 2025. Prior to the survey, the research team communicated with institutional management to explain research objectives and procedures and to secure institutional cooperation. The survey was administered through face-to-face interviews, in which trained interviewers posed each question sequentially and recorded responses. All interviewers were graduate students in public health or social work who had received standardized training prior to formal data collection, covering research ethics, questionnaire interpretation, and interviewing techniques.

The following quality control measures were implemented: (1) questionnaire items used concise, easy-to-understand language and avoided technical terminology; (2) interviewers provided appropriate explanations during interviews according to older adults' comprehension ability; (3) each completed questionnaire was reviewed on-site by the interviewer to ensure no missing items; and (4) the research supervisor spot-checked 10% of questionnaires daily, with immediate correction of any identified issues.

During the survey period, 347 eligible older adults were approached, of whom 35 were excluded due to poor health status or failure to meet cognitive screening criteria. A total of 312 older adults completed the questionnaire. After data cleaning, 34 invalid questionnaires were excluded (24 incomplete responses and 10 with logical inconsistencies), yielding 278 valid questionnaires (effective response rate: 89.1%).

### 2.4 Measurement instruments

A self-developed “Questionnaire on Sports Service Demand and Satisfaction of Older Adults in residential care facilities” was employed. The questionnaire design referenced the SERVQUAL scale structure and drew on item content from relevant domestic research (9, 25). After expert review, pilot testing, and reliability and validity testing, the final questionnaire comprised three sections.

The demographic section assessed participant and institutional characteristics through 12 items, including gender, age, education level, monthly income, marital status, length of stay, self-rated health status, number of chronic diseases, self-care ability, institution type, institution size, and reason for admission. Self-rated health status was measured on a 5-point scale (1 = very poor, 5 = very good), and self-care ability was classified into three categories based on the Activities of Daily Living (ADL) scale: “fully independent,” “partially independent,” and “needs care.”

The demand scale comprised 20 items across five dimensions with four items each. Facilities items covered indoor activity room, outdoor activity space, age-friendly fitness equipment, and barrier-free facilities. Fitness guidance items included professional fitness instructor, individualized exercise prescription, exercise safety protection, and rehabilitation training guidance. Activity organization items covered group fitness activities, small-group fitness activities, intergenerational interaction activities, and festival sports activities. Information consultation items encompassed fitness knowledge publicity, exercise effect evaluation feedback, fitness method consultation, and fitness information delivery. Physical monitoring items covered regular physical fitness testing, chronic disease exercise monitoring, exercise data recording, and health risk warning. All demand items employed a 5-point Likert scale (1 = not needed at all, 2 = not much needed, 3 = somewhat needed, 4 = quite needed, 5 = very much needed), with higher scores indicating stronger demand.

The satisfaction scale employed a parallel structure with 20 items corresponding one-to-one with the demand scale, using a 5-point Likert scale (1 = very dissatisfied, 2 = somewhat dissatisfied, 3 = neutral, 4 = somewhat satisfied, 5 = very satisfied). According to SERVQUAL theoretical logic, satisfaction as older adults' perceptual evaluation of actual service supply serves as an indirect indicator of service supply level (18).

Scale reliability testing (Table 1) indicated that Cronbach's α coefficients ranged from 0.767 to 0.845 for the demand scale and from 0.784 to 0.837 for the satisfaction scale, all meeting the acceptable standard of above 0.7 and most exceeding the good standard of 0.8, indicating good internal consistency reliability (26).

**Table 1:** Scale reliability testing results.

**Scale**	**Dimension**	**Items**	**Cronbach's α**
Demand scale	Facilities	4	0.822
	Fitness guidance	4	0.767
	Activity organization	4	0.816
	Information consultation	4	0.830
	Physical monitoring	4	0.845
	Total scale	20	0.871
Satisfaction scale	Facilities	4	0.812
	Fitness guidance	4	0.798
	Activity organization	4	0.822
	Information consultation	4	0.784
	Physical monitoring	4	0.837
	Total scale	20	0.852

### 2.5 Variable measurement

The dependent variable is the supply-demand matching status, which was operationalized as a binary variable using an item-counting approach (27). The supply side was measured using newly developed service accessibility items (SA1–SA5), which assessed service provision across five dimensions in a binary format (“Does the institution provide this service?” Yes/No): facility accessibility (SA1), fitness guidance accessibility (SA2), activity organization accessibility (SA3), information consultation accessibility (SA4), and physical monitoring accessibility (SA5). The number of “yes” responses was summed to yield the supply count (supply_count, range: 0–5). The demand side retained the original demand scale: the demand mean was computed for each of the five dimensions, and dimensions with means >4.0 were flagged as “urgent needs” (indicating the service was “quite needed” or “very much needed”), yielding the urgent-need dimension count (urgent_count, range: 0–5). The supply-demand gap was defined as: gap = supply_count − urgent_count. When gap ≤ 0, indicating that the number of accessible service dimensions did not exceed the number of urgent-need dimensions, the case was coded as 1 (supply-deficit group); when gap >0, it was coded as 0 (supply-demand balance group). Compared with the original approach using a satisfaction threshold (>3.5) to gauge supply adequacy, the service accessibility items directly capture institution-level objective service provision, reducing the influence of subjective perceptual bias. This operationalization transforms continuous supply-demand information into interpretable integer indicators, capturing the multi-dimensional supply-demand matching status.

*Independent variables* comprised three categories:

(1) Demographic characteristics: gender (male = 1, female = 0), age group (60–69 = 1, 70–79 = 2, 80–89 = 3, ≥90 = 4), education level (primary or below = 1, junior high = 2, senior high or secondary = 3, college or above = 4), and monthly income (<2,000 yuan = 1, 2,000–3,000 = 2, 3,000–5,000 = 3, >5,000 = 4).

(2) Functional status and institutional characteristics: self-care ability (needs care = 1, partially independent = 2, fully independent = 3), length of stay (<1 year = 1, 1–3 years = 2, >3 years = 3), and institution type (with public institutions as the reference category, two dummy variables were created for private and PPP institutions).

(3) Service perception characteristics: overall satisfaction, computed as the mean of all 20 satisfaction items, *Z*-score standardized prior to model entry (*M* = 3.130, *SD* = 0.223). The regression coefficient reflects the effect of each one-standard-deviation increase.

### 2.6 Statistical analysis

Data were analyzed using SPSS 27.0 (IBM Corp., Armonk, NY, USA). Descriptive statistics included frequencies and percentages for categorical variables, means and standard deviations for continuous variables, and radar charts for visual comparison of demand and satisfaction across dimensions. Supply-demand gap analysis computed per-dimension gaps (demand mean minus satisfaction mean) and shortage rates (gap/demand intensity × 100%) to evaluate relative supply-demand differences and assign priority levels. A 2 × 2 supply-demand matrix was constructed using the medians of demand and satisfaction as boundaries, classifying dimensions into four quadrants for differentiated strategy formulation. Reliability was assessed using Cronbach's α coefficients, with α ≥ 0.7 considered acceptable and α ≥ 0.8 considered good (26). Comparisons of resident characteristics across institution types employed Kruskal–Wallis tests (for continuous and ordinal variables) and chi-square tests (for categorical variables) to assess whether institution type was systematically associated with resident characteristics and service demand.

Binary logistic regression was used to analyze predictors of supply-demand matching. The model included nine predictor variables: demographic characteristics (gender, age, education level, monthly income), functional status (self-care ability, length of stay), institution type (dummy variables with public institutions as reference), and overall satisfaction (*Z*-score standardized). Overall satisfaction was standardized prior to model entry to obtain interpretable effect size estimates. Model significance was evaluated using the likelihood ratio test (LLR χ^2^), explanatory power using Nagelkerke pseudo-*R*^2^, goodness of fit using the Hosmer-Lemeshow test, and predictive performance using classification accuracy. All statistical tests were two-tailed with a significance level of α = 0.05. Bonferroni correction was applied for pairwise *post-hoc* comparisons following significant Kruskal–Wallis tests to control the Type I error rate. Note that ordinal variables (education level and monthly income) were entered as continuous predictors in the logistic regression, which assumes equal-interval effects across categories; the appropriateness of this assumption is discussed as a study limitation.

## 3 Results

### 3.1 Sample characteristics

Table 2 presents the demographic and institutional characteristics of the 278 respondents. Regarding gender distribution, females comprised 55.76% (*n* = 155) and males 44.24% (*n* = 123), yielding a male-to-female ratio of approximately 1:1.26. The age distribution was dominated by middle-old and oldest-old groups: the 70–79 age group had the highest proportion (42.81%, *n* = 119), followed by 80–89 (30.58%, *n* = 85), 60–69 (19.06%, *n* = 53), and ≥90 (7.55%, *n* = 21). Older adults aged 80 and above combined accounted for approximately 38%, reflecting that residential care facility residents are predominantly in the middle-old to oldest-old age groups. Education levels were generally low: primary school or below accounted for 48.92% (*n* = 136), junior high school for 28.42% (*n* = 79), senior high/secondary for 16.55% (*n* = 46), and college or above for only 6.12% (*n* = 17). This distribution reflects the generational characteristics of current institutional residents—most were born in the 1930s–1950s, when educational opportunities were limited. Monthly income was relatively evenly distributed: 3,000–5,000 yuan (30.58%, *n* = 85), 2,000–3,000 yuan (28.78%, *n* = 80), below 2,000 yuan (25.54%, *n* = 71), and above 5,000 yuan (15.11%, *n* = 42). Regarding self-care ability, 48.92% (*n* = 136) needed care, 25.18% (*n* = 70) were partially independent, and 25.90% (*n* = 72) were fully independent. Nearly half needed care, reflecting the high degree of functional dependence among institutional residents. Length of stay was distributed as follows: 1–3 years (48.92%, *n* = 136), over 3 years (29.14%, *n* = 81), and under 1 year (21.94%, *n* = 61). Institution types were represented as: public (41.01%, *n* = 114), private (33.09%, *n* = 92), and PPP (25.90%, *n* = 72).

**Table 2:** Demographic characteristics of respondents (*N* = 278).

**Variable**	**Category**	***n***	**%**
Gender	Female	155	55.76
	Male	123	44.24
Age	60–69	53	19.06
	70–79	119	42.81
	80–89	85	30.58
	≥90	21	7.55
Education	Primary or below	136	48.92
	Junior high	79	28.42
	Senior high/Secondary	46	16.55
	College or above	17	6.12
Monthly income	<2,000 yuan	71	25.54
	2,000–3,000 yuan	80	28.78
	3,000–5,000 yuan	85	30.58
	>5,000 yuan	42	15.11
Self-care ability	Needs care	136	48.92
	Partially independent	70	25.18
	Fully independent	72	25.90
Length of stay	<1 year	61	21.94
	1–3 years	136	48.92
	>3 years	81	29.14
Institution type	Public	114	41.01
	Private	92	33.09
	Public-private partnership	72	25.90

### 3.2 Comparison of resident characteristics across institution types

Resident demographics and service demand were compared across institution types (Table 3).

**Table 3:** Comparison of resident characteristics across institution types (*N* = 278).

**Variable**	**Public (*n* = 114)**	**Private (*n* = 92)**	**PPP (*n* = 72)**	**Test statistic**	***p***
Self-care ability^*a*^	1.65 (0.85)	1.93 (0.81)	1.75 (0.82)	*H* = 7.243	0.027^*^
Monthly income^*a*^	2.17 (1.03)	2.62 (0.96)	2.31 (1.03)	*H* = 10.595	0.005^**^
Age group^*a*^	2.22 (0.81)	2.35 (0.92)	2.24 (0.85)	*H* = 1.368	0.505
Education level^*a*^	1.78 (0.93)	1.89 (0.93)	1.71 (0.93)	*H* = 2.151	0.341
Self-rated health^*a*^	3.16 (1.05)	3.16 (0.94)	2.88 (1.02)	*H* = 4.233	0.121
Male proportion^*b*^	45.6%	41.3%	45.8%	χ^2^ = 0.483	0.786
Overall satisfaction^*a*^	3.139 (0.23)	3.123 (0.21)	3.126 (0.24)	*H* = 0.726	0.695
Supply-demand gap rate^*b*^	36.8%	31.5%	36.1%	χ^2^ = 0.698	0.705

^*a*^Kruskal–Wallis test, reporting mean (SD); ^*b*^Chi-square test, reporting percentage.

^**^p < 0.01, ^*^p < 0.05.

Significant differences were observed across institution types in demographic characteristics. Public institution residents had significantly lower self-care ability (*M* = 1.65 vs. 1.93, *p* = 0.027) and monthly income (*M* = 2.17 vs. 2.62, *p* = 0.005) compared with private institution residents, whereas no significant differences emerged for age, education level, self-rated health, or gender distribution. These findings support the “safety net” function of public institutions—compared with private and PPP institutions, public institutions concentrate more residents with limited self-care ability and economic vulnerability. This is consistent with China's care policy for older adults design, whereby public institutions prioritize admission of “three-no” older adults (those without income, family support, or working ability) and low-income disabled older adults (28).

Notably, despite significant differences in resident characteristics, overall satisfaction (public *M* = 3.139, private *M* = 3.123, PPP *M* = 3.126; *H* = 0.726, *p* = 0.695) and supply-demand gap rates (public 36.8%, private 31.5%, PPP 36.1%; χ^2^ = 0.698, *p* = 0.705) did not differ significantly across institution types. This pattern of “different residents, similar service perceptions” suggests that institution type per se may not be a decisive factor in supply-demand matching; the specific mechanisms underlying this pattern are further examined in the subsequent regression analysis.

### 3.3 Sports service supply-demand status

#### 3.3.1 Demand intensity analysis

Table 4 presents demand intensity and satisfaction scores across the five dimensions. Fitness guidance had the highest demand (*M* = 4.05, *SD* = 0.44), falling between “quite needed” and “very much needed”; facilities followed closely (*M* = 4.03, *SD* = 0.42), also at a high demand level. Both dimensions had demand means exceeding 4.0, indicating strong demand among institutional older adults for professional fitness guidance and age-friendly facilities.

**Table 4:** Demand and satisfaction by service dimension (*N* = 278).

**Dimension**	**Demand *M***	**Demand SD**	**Satisfaction *M***	**Satisfaction SD**
Facilities	4.03	0.42	3.00	0.60
Fitness guidance	4.05	0.44	3.03	0.57
Activity organization	3.57	0.51	3.30	0.61
Information consultation	3.51	0.56	3.19	0.62
Physical monitoring	3.48	0.62	3.21	0.63

Activity organization had moderate demand (*M* = 3.57, *SD* = 0.51), falling between “somewhat needed” and “quite needed.” Information consultation (*M* = 3.51, *SD* = 0.56) and physical monitoring (*M* = 3.48, *SD* = 0.62) had relatively lower demand, though still at an above-moderate level. Demand intensity was ranked as: fitness guidance > facilities > activity organization > information consultation > physical monitoring.

From a practical standpoint, this ranking reflects the demand priorities of institutional residents. Fitness guidance and facilities represent basic prerequisites for engaging in physical activity, serving as preconditions for achieving higher-level fitness goals and thus receiving priority consideration from older adults. However, demand intensity may also be influenced by the availability of services—dimensions with lower current supply may elicit stronger expressed demand due to unmet needs, rather than solely reflecting an intrinsic hierarchical ordering. Activity organization represents social participation needs, fulfilling expectations for group activities and social interaction. Information consultation and physical monitoring constitute developmental needs that may receive greater attention once basic service needs are met.

#### 3.3.2 Satisfaction analysis

Regarding satisfaction (perceived supply), activity organization received the highest rating (*M* = 3.30, *SD* = 0.61), falling between “neutral” and “somewhat satisfied”; physical monitoring (*M* = 3.21, *SD* = 0.63) and information consultation (*M* = 3.19, *SD* = 0.62) followed; fitness guidance (*M* = 3.03, *SD* = 0.57) and facilities (*M* = 3.00, *SD* = 0.60) received the lowest satisfaction, reaching only the “neutral” level. The satisfaction ranking across dimensions was: activity organization > physical monitoring > information consultation > fitness guidance > facilities.

This ranking reflects the structural characteristics of current sports service supply in residential care facilities. Activity organization services (such as tai chi and square dancing group activities), due to their low cost and ease of organization, are relatively common in residential care facilities, resulting in relatively higher satisfaction. In contrast, facility renovation and professional fitness guidance services, requiring larger capital investment and professional talent support, have relatively insufficient supply and lower satisfaction.

#### 3.3.3 Supply-demand gap analysis

Table 5 presents the supply-demand gap and related indicators across the five dimensions. All dimensions exhibited positive gaps, indicating an overall supply deficit in institutional sports services. Facilities had the largest gap (1.03), with a shortage rate of 25.6%, meaning actual supply met only approximately 74% of demand; fitness guidance followed closely (1.02), with a shortage rate of 25.2%. These two dimensions represent high-priority areas requiring preferential resource allocation.

**Table 5:** Supply-demand gap analysis.

**Dimension**	**Demand**	**Satisfaction**	**Gap**	**Shortage rate**	**Priority**
Facilities	4.03	3.00	1.03	25.6%	High
Fitness guidance	4.05	3.03	1.02	25.2%	High
Information consultation	3.51	3.19	0.32	9.1%	Low
Activity organization	3.57	3.30	0.27	7.6%	Low
Physical monitoring	3.48	3.21	0.27	7.8%	Low

Gap = demand mean − satisfaction mean. Shortage rate = gap/demand mean × 100%. Gap values were computed from unrounded means; minor discrepancies with table values are due to rounding.

Information consultation had a gap of 0.32, with a shortage rate of 9.1%; activity organization and physical monitoring had the smallest gaps (both 0.27), with shortage rates of 7.6 and 7.8%, respectively, indicating relatively better supply-demand matching. These dimensions can be classified as lower-priority areas where maintaining current service levels is appropriate under resource constraints.

Notably, the ranking of demand intensity was strikingly inverted relative to satisfaction ranking. The two dimensions with the most urgent demand—facilities and fitness guidance—had the lowest satisfaction, whereas activity organization, with the highest satisfaction, ranked only third in demand intensity. This structural contradiction of “high demand-low supply” coexisting with “low demand-high supply” profoundly reveals the supply-demand misallocation problem in current residential care facility sports service resource allocation.

From a service economics perspective, this mismatch reflects the fundamental tension between supply-driven and demand-driven approaches. Most residential care facility currently follow a “cost minimization” logic in sports service provision—prioritizing low-cost, easily implementable services (such as group activities) while neglecting high-cost but urgently needed services (such as professional guidance and facility construction). Although this supply model saves operational costs in the short term, it produces severe misalignment between resource allocation and demand structure, reducing the overall effectiveness of service provision. This supply-demand mismatch phenomenon has been widely observed in China's care service sector for older adults, where government-invested facilities often fail to match actual user needs (29).

#### 3.3.4 Specific item supply-demand gap

Table 6 lists the top 10 specific service items by supply-demand gap, representing areas most urgently needing improvement for older adults. Barrier-free facilities ranked first (supply-demand gap = 1.44), with demand mean as high as 4.25 but satisfaction only 2.81, and a shortage rate as high as 33.9%. This result indicates obvious deficiencies in age-friendly renovation of residential care facilities, failing to meet the basic needs of advanced-age and disabled older adults for safe participation in physical activities.

**Table 6:** Top 10 items by supply-demand gap.

**Rank**	**Dimension**	**Item**	**Demand**	**Satisfaction**	**Gap**
1	Facilities	Barrier-free facilities	4.25	2.81	1.44
2	Fitness guidance	Safety protection	4.12	2.92	1.20
3	Facilities	Age-friendly equipment	3.99	2.87	1.12
4	Facilities	Indoor activity room	4.13	3.06	1.06
5	Fitness guidance	Exercise prescription	3.94	2.90	1.04
6	Fitness guidance	Professional instructor	4.15	3.13	1.02
7	Fitness guidance	Rehabilitation training	4.12	3.22	0.90
8	Info. consultation	Effect evaluation	3.64	2.93	0.71
9	Physical monitoring	Chronic disease monitoring	3.60	2.92	0.67
10	Facilities	Outdoor space	3.94	3.28	0.66

Items ranked second through sixth were: safety protection (supply-demand gap = 1.20), age-friendly equipment (1.12), indoor activity room (1.06), exercise prescription (1.04), and professional instructor (1.02). These items share two common characteristics: first, they all involve basic conditions for older adults to participate in physical activities, including safety assurance (barrier-free facilities, safety protection), equipment conditions (age-friendly equipment, indoor activity room), and professional guidance (exercise prescription, professional instructor); second, they all require higher levels of professionalization and capital investment, not easily solved by simply adding staff or organizing activities.

Rehabilitation training (supply-demand gap = 0.90), effect evaluation (0.71), chronic disease monitoring (0.67), and outdoor space (0.66) ranked seventh through tenth. Although their supply-demand gaps were relatively smaller, they still represent areas needing improvement. Particularly rehabilitation training has special importance for the high proportion of disabled and semi-disabled older adults in older adults care institutions.

#### 3.3.5 Service accessibility analysis

Table 7 presents service accessibility (i.e., the proportion of institutions providing each service) overall and by institution type. Activity organization had the highest accessibility (79.1% overall), with public institutions reaching 83.3%, private 72.8%, and PPP 80.6%. Information consultation (61.9%) and physical monitoring (51.8%) had moderate accessibility. Facility accessibility (44.6%) and fitness guidance accessibility (34.5%) were the lowest, with fewer than half of respondents' institutions providing these services, consistent with the supply-demand gap analysis above.

**Table 7:** Service accessibility by dimension (%).

**Dimension**	**Overall (*N* = 278)**	**Public (*n* = 114)**	**Private (*n* = 92)**	**PPP (*n* = 72)**
Facilities	44.6	48.2	46.7	36.1
Fitness guidance	34.5	34.2	34.8	34.7
Activity organization	79.1	83.3	72.8	80.6
Information consultation	61.9	60.5	55.4	72.2
Physical monitoring	51.8	59.6	45.7	47.2

Values represent the percentage of respondents reporting “institution provides this service.”

Across institution types, service accessibility patterns were broadly similar, with localized differences. Public institutions had the highest accessibility for activity organization (83.3%) and physical monitoring (59.6%); PPP institutions led in information consultation (72.2%); while facilities and fitness guidance remained at low levels across all three types. Fitness guidance accessibility was virtually identical across institution types (34.2%–34.8%), indicating that the shortage of professional fitness guidance personnel is a systemic challenge common to all types of older adults care institutions.

#### 3.3.6 Supply-demand gap distribution

Table 8 presents the frequency distribution of the supply-demand gap (supply_count − urgent_count). Gap values ranged from −2 to +5, with negative values indicating more urgent-need dimensions than accessible service dimensions (supply deficit) and positive values indicating the converse. Respondents with a gap ≤ 0 totaled 97 (34.9%), coded as the supply-deficit group; those with a gap > 0 totaled 181 (65.1%), coded as the supply-demand balance group. The distribution was right-skewed, with a mode of +1 (*n* = 79, 28.4%) and median of +1, indicating that most respondents had slightly more accessible service dimensions than urgent-need dimensions.

**Table 8:** Supply-demand gap distribution (*N* = 278).

**Gap value**	**Frequency**	**Percentage (%)**
−2	4	1.4
−1	27	9.7
0	66	23.7
+1	79	28.4
+2	52	18.7
+3	35	12.6
+4	12	4.3
+5	3	1.1

Gap = supply_count − urgent-need dimension count (urgent_count).

Gap ≤ 0: supply-deficit group (97, 34.9%); gap > 0: supply-demand balance group (181, 65.1%).

### 3.4 Supply-demand two-dimensional coupling analysis

Using demand median (3.57) and satisfaction median (3.19) as boundaries, a supply-demand two-dimensional analysis matrix was constructed (Table 9). This matrix classifies the five service dimensions into four quadrants, providing a basis for differentiated strategy formulation.

**Table 9:** Sports service supply-demand two-dimensional analysis matrix.

**Dimension**	**Demand**	**Satisfaction**	**Quadrant**	**Strategy**
Facilities	4.03	3.00	I (Urgent improvement)	Priority investment
Fitness guidance	4.05	3.03	I (Urgent improvement)	Priority investment
Activity organization	3.57	3.30	II (Strength maintenance)	Maintain and optimize
Information consultation	3.51	3.19	IV (Resource reallocation)	Moderate adjustment
Physical monitoring	3.48	3.21	IV (Resource reallocation)	Moderate adjustment

Quadrant I (high demand-low satisfaction): includes facilities and fitness guidance dimensions, representing areas with the most prominent supply-demand contradictions. These services are characterized by urgent demand from older adults but inadequate current supply levels. From a resource allocation perspective, these two dimensions should be priority improvement targets, with increased investment through facility construction, talent development, and service innovation to raise supply levels.

Quadrant II (high demand-high satisfaction): includes only activity organization. These services have relatively high demand and relatively adequate supply, with relatively good supply-demand matching, representing relative strength areas for institutions. The strategy recommendation is to maintain current service levels, continuously optimize while ensuring service quality, and consider reallocating some resources to Quadrant I areas.

Quadrant III (low demand-low satisfaction): no dimensions fell into this quadrant in this study. If such services exist, their necessity usually needs evaluation, or service content redesign is required.

Quadrant IV (low demand-high satisfaction): includes information consultation and physical monitoring dimensions. Current supply levels for these services exceed older adults' demand intensity, with some degree of resource over-allocation. The strategy recommendation is to moderately adjust resource investment, reallocating some resources to Quadrant *I*'s urgent improvement areas to achieve Pareto optimization. It should be noted that this does not mean weakening these services, but optimizing resource allocation structure while ensuring basic service quality.

### 3.5 Predictors of supply-demand matching

Table 10 presents the binary logistic regression results. The model included nine predictor variables (demographic characteristics, functional status, overall satisfaction, and institution type) and yielded a Nagelkerke pseudo-*R*^2^ of 0.223, indicating moderate explanatory power. The likelihood ratio test (LLR χ^2^ = 49.06, *df* = 9, *p* < 0.001) confirmed overall model significance. Among 278 respondents, 97 were classified in the supply-deficit group (DV = 1, 34.9%) and 181 in the supply-demand balance group (DV = 0, 65.1%). The Hosmer–Lemeshow test (χ^2^ = 3.071, *p* = 0.930) indicated adequate model fit, with a classification accuracy of 69.1%.

**Table 10:** Logistic regression results for predictors of supply-demand matching (*N* = 278).

**Variable**	***B***	**SE**	**Wald**	**OR**	**95% CI**	***p***
Constant	1.482	0.769	3.712	4.401	—	0.054
Gender (male = 1)	−0.092	0.278	0.109	0.912	0.529–1.574	0.742
Age group	0.050	0.164	0.092	1.051	0.762–1.449	0.762
Education level	−0.491	0.159	9.527	0.612^**^	0.448–0.836	0.002
Monthly income	−0.503	0.139	13.055	0.604^***^	0.460–0.794	<0.001
Self-care ability	−0.095	0.167	0.322	0.910	0.656–1.261	0.571
Length of stay	0.001	0.195	0.000	1.001	0.683–1.465	0.997
Overall satisfaction^*b*^	−0.725	0.157	21.186	0.484^***^	0.356–0.660	<0.001
Private institution^*a*^	−0.222	0.330	0.454	0.801	0.420–1.528	0.500
PPP institution^*a*^	−0.081	0.345	0.056	0.922	0.469–1.811	0.813

Model fit: −2*LL* = 310.55, LLR χ^2^ = 49.06 (*df* = 9, *p* < 0.001), Nagelkerke *R*^2^ = 0.223.

Hosmer-Lemeshow test: χ^2^ = 3.071, *p* = 0.930; classification accuracy = 69.1%.

^***^p < 0.001, ^**^p < 0.01.

^*a*^Institution type reference category = public.

^*b*^Overall satisfaction *Z*-score standardized (*M* = 3.130, *SD* = 0.223); OR reflects the effect per one-SD increase.

Supply-deficit group (DV = 1): 97 (34.9%); supply-demand balance group (DV = 0): 181 (65.1%).

Overall satisfaction was the strongest predictor of supply-demand matching (OR = 0.484, *p* < 0.001), indicating that after controlling for other variables, each one-standard-deviation increase in satisfaction was associated with a 51.6% reduction in the odds of supply deficit. This result indicates that older adults with higher perceived service quality were less likely to be classified in the supply-deficit group. From a SERVQUAL theoretical perspective, satisfaction reflects a comprehensive evaluation of overall service quality; higher satisfaction implies that the perceived service provision level is closer to meeting needs, resulting in a smaller subjective gap between supply and demand and thus a lower likelihood of supply-demand imbalance. Marginal effects analysis showed that each one-standard-deviation increase in satisfaction reduced the probability of supply deficit by 13.7 percentage points (ME = −0.137, *p* < 0.001), further confirming the substantive impact of satisfaction.

Education level significantly predicted supply-demand matching (OR = 0.612, *p* = 0.002), with each one-level increase in education associated with an approximately 39% reduction in the odds of supply deficit. Note that education level was entered as an ordinal variable (coded 1–4), which assumes equal-interval effects across levels; this assumption is discussed as a limitation. This finding can be explained through differences in information access and service utilization capacity: older adults with higher education levels typically possess stronger abilities to search for and comprehend information, enabling more effective access to and utilization of various sports service resources provided by the institution. Subgroup analysis revealed that the supply-deficit rate was 44.1% among those with primary education or below, compared with 26.6% (junior high), 26.1% (senior high/secondary), and 23.5% (college or above), demonstrating a clear gradient of decline. Marginal effects analysis indicated that each one-level increase in education reduced the probability of supply deficit by 9.3 percentage points (ME = −0.093, *p* = 0.001).

Monthly income was also a significant predictor (OR = 0.604, *p* < 0.001), with each one-level increase in income associated with an approximately 40% reduction in the odds of supply deficit. The association between economic resources and supply-demand matching may operate through multiple pathways: higher-income older adults may have greater capacity in selecting care institutions and may be more likely to reside in institutions with more comprehensive service configurations; additionally, supplementary sports services in some institutions require extra payment, which more affluent residents can better afford. However, given the cross-sectional design, these pathways remain speculative and warrant verification through longitudinal research. Subgroup analysis revealed supply-deficit rates of 46.5% (<2,000 yuan), 41.2% (2,000–3,000), 30.6% (3,000–5,000), and 11.9% (>5,000), demonstrating a pronounced income gradient. Marginal effects analysis showed that each one-level increase in income reduced the probability of supply deficit by 9.5 percentage points (ME = −0.095, *p* < 0.001).

Self-care ability (OR = 0.910, *p* = 0.571), gender (OR = 0.912, *p* = 0.742), age (OR = 1.051, *p* = 0.762), and length of stay (OR = 1.001, *p* = 0.997) were all non-significant in the model (all *p* > 0.05). Notably, after controlling for satisfaction and socioeconomic variables, institution type was also non-significant (private OR = 0.801, *p* = 0.500; PPP OR = 0.922, *p* = 0.813). Combined with the findings from Table 3—that public institution residents had significantly lower self-care ability and monthly income than private institution residents, yet satisfaction and supply-demand gap rates did not differ significantly across institution types—the non-significant effect of institution type on supply-demand matching is consistent with a compositional effect: public institutions concentrate more functionally impaired and economically vulnerable older adults, fulfilling a social safety-net function, but this structural difference in resident composition was not associated with significant differences in sports service supply-demand matching. This finding suggests that supply-demand matching is primarily associated with individual-level service perceptions and socioeconomic characteristics rather than institution-level structural factors, although the possibility of insufficient statistical power to detect moderate institution-type effects cannot be excluded.

## 4 Discussion

### 4.1 Structural imbalance: cost-driven resource misallocation

This study revealed a pronounced structural imbalance in the supply and demand of sports services in residential care facilities. The supply–demand gaps for facilities and fitness instruction were 1.03 and 1.02, respectively (shortage rates exceeding 25%), whereas the gap for activity organization was merely 0.27 (shortage rate 7.6%). Demand intensity ranking was strikingly inverted relative to satisfaction ranking—the dimensions with the most urgent demand (facilities and fitness instruction) had the lowest satisfaction, while activity organization, with the highest satisfaction, ranked only third in demand, presenting a structural contradiction of “high demand–low supply” coexisting with “low demand–high supply.” The root cause of this differentiation lies in disparities in service cost structure. Activity organization (e.g., tai chi and square dancing) requires only low-marginal-cost coordination and is preferentially supplied under a “cost-minimization” logic (29). Service accessibility data corroborate this mechanism: activity organization accessibility reached 79.1%, compared with only 44.6% for facilities and 34.5% for fitness instruction. Moreover, fitness instruction accessibility was virtually identical across all three facility types (34.2%–34.8%), indicating that the shortage of professional personnel is a systemic problem rather than a management failure of individual facilities. Notably, the facilities dimension encompasses items such as barrier-free facilities, age-appropriate equipment, and safety protection—without these basic accessibility and safety conditions, higher-level exercise participation cannot be realized. This aligns with a systematic review that identified environmental constraints, insufficient professional guidance, and fear of injury as the three primary barriers to physical activity among adults aged 70 and above (30). Situated within the broader context of Chinese older adults care research, this supply–demand mismatch is not an isolated phenomenon. Similar structural imbalances have been identified at the community level (8, 9). However, the contradiction in the institutional context possesses fundamentally distinct characteristics rooted in the systematic differences between the two populations. Community-dwelling older adults largely retain autonomous exercise capacity, with their core challenge being spatial accessibility; in contrast, 48.92% of respondents in the present study required care (impaired self-care ability), with substantially higher rates of advanced age and multi-morbidity compared with community populations. This directly explains why facility-related services—particularly those addressing accessibility and safety needs—exhibited the largest supply–demand gaps in institutional settings yet are typically unremarkable in community-based studies (31, 32). In other words, the health vulnerability of institutional residents amplifies “safety-assurance” needs to the highest priority, while the current institutional supply system exhibits its largest deficits precisely in these dimensions—the mismatch is not merely insufficient service quantity but a systematic deviation between service types and residents' functional profiles (33, 34).

### 4.2 Individual-level determinants and compositional effects

Logistic regression identified three significant predictors of supply–demand mismatch. Overall satisfaction was the strongest predictor (OR = 0.484, *p* < 0.001), with each one-standard-deviation increase associated with a 51.6% reduction in the odds of supply deficit (ME = −0.137, *p* < 0.001). From a SERVQUAL theoretical perspective, satisfaction reflects “perceived service quality,” integrating multi-dimensional evaluations of responsiveness, reliability, and tangibles, thereby systematically predicting supply–demand matching status (18). However, two methodological caveats merit attention. First, a “satisfaction paradox” observed in public service research warrants cautious interpretation—low-expectation groups may report higher satisfaction even when facing equivalent objective supply, owing to lower reference standards (35). In the present study, although public facility residents had significantly lower self-care ability and income, their satisfaction did not differ significantly from that of private and PPP facility residents (*p* = 0.695), suggesting that expectation-level differences may play a moderating role. Education level (OR = 0.612, *p* = 0.002) and monthly income (OR = 0.604, *p* < 0.001) constituted a dual socioeconomic barrier. The influence of education can be explained through a health literacy mechanism: older adults with higher education possess stronger information-seeking and comprehension abilities, enabling more effective identification and utilization of sports service resources within their facilities. This is consistent with a systematic review demonstrating that insufficient health literacy is prevalent among older populations, constraining their ability to access, understand, and use health information (36). A study of 740 older adults across 16 medical-nursing integrated facilities in Chengdu, China, further supports this pathway: only 6.33% of institutional older adults possessed basic health literacy, with education level and monthly income as significant predictors (37).The association between income and supply–demand matching may operate through two pathways: facility selection and supplementary service purchasing. Higher-income older adults may be more likely to reside in facilities with comprehensive service configurations and may be better positioned to purchase additional sports service programs. The supply-deficit rate was only 11.9% in the >5,000 CNY group, compared with 46.5% in the <2,000 CNY group—a nearly fourfold disparity. Research on low-income older adults in U.S. senior housing has similarly reported associations between economic limitations and reduced physical activity resource access (38). Within the policy objective of equalizing older adults care services in China, this income gradient suggests that low-income groups may face a “dual economic–informational” disadvantage. However, these pathways are inferred from cross-sectional associations and require confirmation through longitudinal or quasi-experimental designs.

Self-care ability did not reach statistical significance (OR = 0.910, *p* = 0.571), which may be attributable to the general lack of functionally differentiated service provision in institutional sports services (14). Fitness instruction accessibility was virtually identical across all three facility types (34.2%–34.8%), indicating that service provision is not stratified by residents' functional needs. Under this “undifferentiated supply” model, functional status differences may not translate into supply–demand matching differences (39). One speculative explanation is a “dual-floor offsetting effect”: functionally impaired residents may objectively have greater need for sports services, yet their expressed demand could be suppressed by diminished health confidence and reduced participation motivation (12). Research has found that physical activity levels across the entire sample in nursing facilities are uniformly extremely low, which may prevent individual differences from manifesting (40). If both demand and accessibility among functionally impaired residents remain simultaneously low, this could drive the net effect of self-care ability toward zero.

The role of facility type was examined as a potential compositional effect through a two-step analysis. First, descriptive comparisons showed that public facility residents had significantly lower self-care ability (*M* = 1.65 vs. 1.93, *p* = 0.027) and monthly income (*M* = 2.17 vs. 2.62, *p* = 0.005) than private facility residents, consistent with the safety-net function of public facilities; however, satisfaction (*p* = 0.695) and supply–demand gap rates (*p* = 0.705) did not differ significantly across the three facility types (28). Second, after incorporating individual characteristics into the regression model, facility type effects became non-significant (private OR = 0.801, *p* = 0.500; PPP OR = 0.922, *p* = 0.813). This two-step pattern is consistent with a compositional effect: observed differences between public and private facilities may be attributable to “who lives there” rather than “how it operates” (41). However, an alternative explanation—that the sample sizes per facility type (114, 92, and 72) may have been insufficient to detect moderate facility-type effects—cannot be ruled out. The statistical power to detect small-to-moderate effects with these subgroup sizes is limited, and the non-significant result should therefore be interpreted as absence of evidence rather than evidence of absence. Nevertheless, this pattern suggests that efforts to improve supply–demand matching should prioritize individual-level service perceptions and socioeconomic barriers, while future research with larger samples should further investigate potential facility-level effects.

### 4.3 International comparison and institutional barriers

Situating the present findings within the international context facilitates identification of the institutional roots of supply–demand imbalance, although direct cross-national comparisons should be interpreted with caution given substantial differences in economic development levels, long-term care insurance systems, and professional workforce structures. The fitness instruction accessibility of only 34.5% in this study contrasts with the institutionalized coverage reported in several developed countries. Japan has incorporated exercise rehabilitation into statutory services through its long-term care insurance, establishing “functional training instructor” positions in care facilities (42). The U.S. federal quality certification framework mandates that nursing facilities provide restorative care (43). Swedish care facilities employ person-centered interventions to improve residents' functional status (44). A network meta-analysis has further confirmed that exercise interventions in institutional settings significantly improve physical function, with an optimal exercise dose of approximately 170 minutes per week (45).The common feature of these international approaches—integrating sports services into standardized care protocols with professional support—is precisely the element most lacking in the Chinese sample of this study. The institutional barriers underlying this gap are manifested at three levels. First, the occupational system gap: China has not yet established a professional exercise guidance position in care facilities analogous to Japan's “functional training instructor,” and the training system for care workers lacks systematic exercise guidance modules (28). Second, financing mechanism deficiencies: China's long-term care insurance remains in the pilot phase, sports services are not included in standardized service packages, and facilities lack stable funding sources for facility renovation and staffing (28). Third, regulatory standard gaps: current service standards for care facilities are centered on basic living assistance and medical nursing, with sports services not yet incorporated into quality assessment and rating systems (46). These three institutional barriers collectively explain the “high demand–low supply” structural contradiction observed in this study. Although China has promoted “integrated medical and nursing care” and healthy aging at the policy level, a significant gap persists between policy texts and service practices at the facility level (4).

### 4.4 Practical implications

Based on the empirical findings above, the following targeted recommendations are proposed, with each recommendation annotated by data source to distinguish direct evidence from literature-based inferences.First, priority investment in barrier-free facility renovation and professional fitness instruction deployment is warranted. Barrier-free facility renovation in care facilities should be incorporated into the reimbursement scope of the long-term care insurance pilot or supported through dedicated fiscal subsidies, with priority given to public facilities (47). Given the universally low accessibility of fitness instruction, it is recommended that exercise guidance be incorporated into the national vocational training standards for care workers. Second, a routine supply–demand gap assessment mechanism based on SERVQUAL gap analysis should be established, with supply–demand gaps measured at least annually and resource allocation priorities dynamically adjusted based on gap rankings (48). Third, barriers to service utilization among low-education and low-income groups should be reduced. This includes developing pictorial, low-literacy sports service information booklets with one-on-one explanations by care staff to enhance service awareness among less-educated residents, waiving supplementary sports service fees for low-income residents to ensure the universality of basic sports services. Fourth, the latent needs of functionally impaired residents should be proactively identified. Exercise participation barriers among functionally impaired individuals primarily stem from environmental constraints and fear of injury, and providing rehabilitation-oriented adaptive training and safety assurance is an effective approach to overcoming these barriers (30).

### 4.5 Limitations

This study has several limitations. First, the cross-sectional design precludes causal inference; the associations identified between satisfaction, education, income, and supply–demand matching should not be interpreted as causal relationships. Longitudinal or quasi-experimental designs would strengthen causal claims. Second, the Zhengzhou sample limits generalizability; multi-regional studies would enhance external validity. Third, facility-level variables (e.g., size, staffing ratios) were not collected; multilevel modeling would better disentangle individual and facility effects. The sample sizes per facility type (114, 92, and 72) may have been insufficient to detect moderate facility-type effects, and the non-significant result for facility type should be interpreted as absence of evidence rather than evidence of absence. Fourth, supply information was derived from resident perceptions; multi-source designs incorporating facility records would provide more comprehensive measurement. Fifth, the model's Nagelkerke *R*^2^ of 0.223, while confirming overall model significance via the likelihood ratio test (LLR χ^2^ = 49.06, *p* < 0.001), indicates moderate-to-low explanatory power, and the classification accuracy of 69.1% represents only a modest improvement over the baseline rate of 65.1%, suggesting that unmeasured factors—such as management philosophy, staffing configurations, and regional policy environments—also influence supply–demand matching. Future research should incorporate additional facility-level and institutional-level variables to enhance model explanatory power.

## 5 Conclusion

This cross-sectional study of older adults in residential care facilities in Zhengzhou revealed significant structural imbalances between the supply of and demand for sports services. Facilities and fitness instruction emerged as the most undersupplied domains, with deficit rates exceeding one quarter, whereas activity organization was relatively well supplied, resulting in a coexisting pattern of “high demand–low supply” and “low demand–high supply.” Satisfaction, education level, and monthly income—rather than functional independence or facility type—were identified as the primary determinants of supply–demand mismatch, constituting a tripartite driving mechanism of “service perception–human capital–economic resources”: higher satisfaction was associated with a smaller perceived gap between service supply and demand; better-educated residents leveraged informational advantages to make more effective use of available services; and higher-income residents secured more adequate provision through facility selection and the purchase of supplementary services. Although the resident profiles of public, private, and public–private partnership facilities differed markedly, the non-significant effect of facility type points to a compositional effect, whereby supply–demand dynamics are driven by individual resident characteristics rather than institutional attributes. These findings underscore the necessity of demand-responsive resource allocation: prioritizing infrastructure development and professional fitness instruction, addressing service accessibility barriers among older adults with lower educational attainment and income, and enhancing residents' awareness of and access to existing services. The analytical framework demonstrated in this study may inform the optimization of sports services in other rapidly aging societies facing similar institutional care challenges.

## Data Availability

The original contributions presented in the study are included in the article/Supplementary material, further inquiries can be directed to the corresponding author.
