# Supplementary material for: Supply-demand structural analysis of sports services in Chinese residential care facilities: a cross-sectional study in Zhengzhou
Source: Front Public Health. 2026 Mar 19;14:1772195. doi: 10.3389/fpubh.2026.1772195 (PMC13043411; doi:10.3389/fpubh.2026.1772195)
Supplement: Supplementary file 1 [file Data_Sheet_1.pdf]

Supplementary Material: Appendix A

Variable Operational Definitions and Coding Scheme

A1. Dependent Variable: Sports Service Supply–Demand Match

Dependent Variable Definition

**Item**	**Description**
Variable name	Sports service supply–demand match status (gap_binary)
Operationalization	Item counting method (following Zhao & Nie, 2023)
**Construction Steps**	
Step 1: Supply side	For each service dimension, determine whether the sports service is accessible/provided. Accessible = 1, Not accessible = 0 (measured directly via service accessibility items SA1–SA5)
Step 2: Demand side	For each service dimension, calculate the mean of 4 demand scale items; classify as "urgent" if mean $>$ 4.0. Urgent = 1, Not urgent = 0
Step 3: Gap calculation	Supply count (accessible dimensions) $-$ Urgent count (urgent demand dimensions)
Step 4: Binary coding	Gap $\leq 0 \rightarrow 1$ (supply insufficiency group); Gap $> 0 \rightarrow 0$ (supply–demand balance group)
Sample distribution	Supply insufficiency: 97 (34.9%); Supply–demand balance: 181 (65.1%)

A2. Core Independent Variable: Overall Sports Service Satisfaction

A3. Control Variable Coding Scheme

A4. Scale Reliability Across Five Service Dimensions

A5. Service Accessibility Questionnaire Items

One item was included for each service dimension to measure the actual accessibility of sports service provision. The item format is as follows:

Core Independent Variable Definition

**Item**	**Description**
Variable name	Overall sports service satisfaction (satisfaction_z)
Measurement	Mean of 20 satisfaction scale items (5 dimensions $\times$ 4 items)
Standardization	Z-score standardization (M = 3.130, SD = 0.223)
Theoretical role	Reflects older adults' perceived evaluation of actual service provision
**Rationale for Selection (Rather Than Demand Intensity)**	
Research design logic	This study aims to examine the predictive role of perceived service quality on supply–demand matching. As the core indicator of "perceived service performance" within the SERVQUAL framework (Parasuraman et al., 1988), satisfaction directly reflects older adults' comprehensive evaluation of service provision levels, making it the predictor most aligned with the theoretical framework
Conceptual independence	Satisfaction is conceptually and operationally independent from both the supply-side "service accessibility" (SA1–SA5) and the demand-side "urgent need" measures, ensuring no definitional overlap between the dependent and independent variables
Methodological precedent	Zhao & Nie (2023) similarly employed satisfaction as the core independent variable in an elderly care service supply–demand matching study (OR = 0.328, $p < 0.05$), validating the feasibility of this approach

Control Variable Coding

Variable	Original Coding	Regression Treatment	Reference
Gender	Male = 1, Female = 0	Binary	Female (ref.)
Age group	60–69 = 1, 70–79 = 2, 80–89 = 3, $\geq$90 = 4	Ordinal continuous	—
Education	Primary or below = 1, Junior high = 2, High school/vocational = 3, College or above = 4	Ordinal continuous	—
Monthly income	$<$2000 CNY = 1, 2000–3000 = 2, 3000–5000 = 3, $>$5000 = 4	Ordinal continuous	—
Self-care ability	Dependent = 1, Partially independent = 2, Fully independent = 3	Ordinal continuous	—
Length of stay	$<$1 year = 1, 1–3 years = 2, $>$3 years = 3	Ordinal continuous	—
Institution type	Public / Private / PPP	2 dummy variables	Public (ref.)

Scale Reliability (Cronbach's α) by Dimension

Dimension	Items	Demand Scale α	Satisfaction Scale α
Facilities & equipment	4	0.822	0.812
Fitness guidance	4	0.767	0.798
Activity organization	4	0.816	0.822
Information & consultation	4	0.830	0.784
Physical fitness monitoring	4	0.845	0.837

Instructions: "Does your elderly care institution currently provide the following sports services to you?" (Yes/No)

Service Accessibility Items

Code	Dimension	Item Content
SA1	Facilities	Does the institution provide indoor/outdoor sports activity areas and fitness equipment?
SA2	Fitness guidance	Does the institution provide professional fitness guidance or exercise prescription services?
SA3	Activity organization	Does the institution organize group fitness activities or sports interest groups?
SA4	Information	Does the institution provide fitness knowledge promotion or exercise consultation services?
SA5	Monitoring	Does the institution provide regular physical fitness testing or chronic disease exercise monitoring?

A6. Logistic Regression Model Specification

Model Specification

**Item**	**Description**
Model type	Binary logistic regression
Dependent variable	Supply–demand match (0 = balance, 1 = insufficiency)
Number of predictors	9 (including 2 dummy variables)
Core independent variable	Overall satisfaction (Z-standardized)
**Model Fit**	
-2 Log Likelihood	310.55
Likelihood ratio test (LR)	χ^2 = 49.06, df = 9, $p < 0.001$
Nagelkerke R^2	0.223
Hosmer–Lemeshow χ^2	3.071 ($p = 0.930$)
Classification accuracy	69.1%
**Key Results**	
Overall satisfaction (Z)	$B = -0.725$, $SE = 0.157$, Wald = 21.186, $OR = 0.484$, 95% CI [0.356, 0.660], $p < 0.001$
Education level	$B = -0.491$, $SE = 0.159$, Wald = 9.527, $OR = 0.612$, 95% CI [0.448, 0.836], $p = 0.002$
Monthly income	$B = -0.503$, $SE = 0.139$, Wald = 13.055, $OR = 0.604$, 95% CI [0.460, 0.794], $p < 0.001$
**Average Marginal Effects**	
Overall satisfaction	dy/dx = -0.137, $p < 0.001$ (absolute probability decrease of approximately 13.7 percentage points)
Education level	dy/dx = -0.093, $p = 0.001$ (absolute probability decrease of approximately 9.3 percentage points)
Monthly income	dy/dx = -0.095, $p < 0.001$ (absolute probability decrease of approximately 9.5 percentage points)
**Interpretation**	
Satisfaction	Each one-standard-deviation increase in satisfaction is associated with approximately 52% lower odds of supply insufficiency
Education	Each one-level increase in education is associated with approximately 39% lower odds of supply insufficiency
Monthly income	Each one-level increase in income is associated with approximately 40% lower odds of supply insufficiency
